# The prognostic and predictive significance of perineural invasion in stage I to III colon cancer: a propensity score matching-based analysis

**DOI:** 10.1186/s12957-024-03405-6

**Published:** 2024-05-11

**Authors:** Chun-Hui Chu, I-Li Lai, Bor-Kang Jong, Sum-Fu Chiang, Wen-Sy Tsai, Pao-Shiu Hsieh, Chien-Yuh Yeh, Jeng-Fu You

**Affiliations:** Division of Colon and Rectal Surgery, Department of Surgery, College of Medicine, Chang Gung Memorial Hospital at Linkou, Chang Gung University, No. 5, Fuxing Street, Guishan District, Taoyuan City, 33305 Taiwan

**Keywords:** Perineural invasion, Colon cancer, Survival, Propensity score matching

## Abstract

**Background:**

Colorectal cancer (CRC) presents with varying prognoses, and identifying factors for predicting metastasis and outcomes is crucial. Perineural invasion (PNI) is a debated prognostic factor for CRC, particularly in stage I-III patients, but its role in guiding adjuvant chemotherapy for node-positive colon cancer remains uncertain.

**Methods:**

We conducted a single-center study using data from the Colorectal Section Tumor Registry Database at Chang Gung Memorial Hospital, Taiwan. This prospective study involved 3,327 CRC patients, 1,536 of whom were eligible after application of the exclusion criteria, to investigate the prognostic value of PNI in stage I-III patients and its predictive value for node-positive/negative cancer patients receiving adjuvant chemotherapy. Propensity score matching (PSM) was used to minimize selection bias, and follow-up was performed with standardized procedures.

**Results:**

PNI-positive (PNI+) tumors were associated with higher preoperative CEA levels and more frequent adjuvant chemotherapy. After PSM, PNI + tumors were associated with marginally significantly lower 5-year disease-free survival (DFS) and significantly lower overall survival (OS) rates in stages III CRC. However, no significant differences were observed in stages I and II. Subgroup analysis showed that among PNI + tumors, only poorly differentiated tumors had higher odds of recurrence. PNI did not predict outcomes in node-negative colon cancer. Adjuvant chemotherapy benefited PNI + patients with node-positive but not those with node-negative disease.

**Conclusions:**

Our study indicates that PNI is an independent poor prognostic factor in stage III colon cancer but does not predict outcomes in node-negative disease. Given the potential adverse effects of adjuvant chemotherapy, our findings discourage its use in node-negative colon cancer when PNI is present.

**Supplementary Information:**

The online version contains supplementary material available at 10.1186/s12957-024-03405-6.

## Introduction

Colorectal cancer (CRC) has been classified as the second most common cause of cancer-related deaths worldwide. Approximately 20% of CRC patients are diagnosed with metastatic disease [1]; further complicating matters, however, 25% of nonmetastatic patients relapse. In nonmetastatic CRC, prognostic data indicate that overall 5-year survival rates are often higher than 50%. In contrast, metastatic CRC is associated with a survival rate of less than 20%. This distinction underscores the importance of discovering the pathophysiological factors underlying the propensity for tumor metastasis. Therefore, understanding these complicated pathophysiological pathways is crucial to improving CRC treatment.

Perineural invasion (PNI) is a distinct pathophysiologic component, setting itself apart from lymphovascular invasions among the various factors contributing to metastasis. However, an ongoing debate surrounds the prognostic value of PNI in stages I to III CRC. Hu et al. contended that PNI lacks substantial prognostic implications for patients with stage I–III CRCs, whereas Leijssen et al. argue the opposite [2, 3]. PNI was found to be a vital predictive factor in a meta-analysis by Knijn et al., especially prominent in patients with nonmetastatic CRC [4]. Regarding specific stages of CRC, Kim et al. categorize PNI as an unfavorable prognostic indicator for recurrence in patients with stage I disease [5], while Mirkin designates it as a negative prognostic indicator for patients with stage II tumors [6]. Suzuki et al., on the other hand, identified PNI as a poor prognostic factor exclusively in stage III CRC patients, with no discernible effects for patients in stages I and II [7].

Several clinicopathological factors have been identified as high-risk elements associated with a poor prognosis in node-negative colon cancer, especially in stage II disease. These factors include T4 tumors, poorly differentiated histology, lymphovascular invasion, PNI, bowel obstruction, tumor perforation, close or positive margins, and insufficiently examined lymph nodes (less than 12). PNI continues to be a contentious predictor of outcomes among these high-risk factors in these patients. According to Enofe et al. and two other meta-analyses [4, 8, 9], PNI is a positive predictive factor for high-risk stage II CRC patients undergoing adjuvant chemotherapy. In contrast, Peng et al. proposed that PNI is not a reliable predictor of a favorable response to adjuvant chemotherapy [10] and considered it an unfavorable characteristic for patients with stage II colon cancer. Leijssen et al. reported in a subgroup analysis of their study [3] that PNI is a prognostic but not predictive factor in nonmetastatic colon cancer. Notably, the patients enrolled in these various investigations exhibited variability in a number of characteristics; for example, rectal cancer patients were included in some studies but not others. Inconsistencies in the included groups may explain these outcomes as rectal cancer treatment options evolve. This highlights the importance of patient demographics and tumor features for interpreting and leveraging prognostic findings for PNI on node-negative colon cancer, especially in the context of adjuvant chemotherapy.

The primary goal of this study was to conduct a thorough analysis of data from a single center, aiming to illuminate the intricate role of PNI in stages I-III colon cancer. Our investigation seeks to delineate how PNI, as a distinctive pathogenic factor, impacts the overall prognosis of patients at different stages of colon cancer. Additionally, our study then broadens its perspective to encompass therapeutic approaches, encompassing both node-positive and node-negative cases of colon cancer. Furthermore, we aimed to assess the predictive accuracy of PNI by examining its influence on the response of colon cancer patients to additional chemotherapy. Our objective is to deepen the understanding of the intricate role that PNI plays in colon cancer through the meticulous analysis and interpretation of our single-center data.

## Materials and methods

The clinicopathological data of patients diagnosed with colorectal cancer were acquired from the Colorectal Section Tumor Registry Database located at Chang Gung Memorial Hospital in Linkou, Taiwan. This information was collected using a prospective approach by a team comprising four nursing specialists who conducted patient interviews and assessed clinicopathological records. These reports were completed using a standardized form during patient admission. The database comprises a comprehensive array of variables, including clinical characteristics, primary complaints, underlying medical conditions, preoperative blood test results, intraoperative factors, postoperative complications, mortality rates, and tumor-related clinicopathological variables. The present investigation obtained ethical approval from the Institutional Review Board (IRB No. 2,311,060,012) at Chang Gung Memorial Hospital.

### Assessment of perineural invasion

At our institution, Chang Gung Memorial Hospital, it is standard practice for pathologists to routinely assess the presence or absence of PNI in all resected CRC specimens. Upon receiving a surgical specimen of a colorectal tumor, the pathology department meticulously examines the tissue sections for various histopathological features, including PNI, as part of the comprehensive pathological evaluation.

The assessment of PNI follows established guidelines and protocols, such as those recommended by the College of American Pathologists (CAP) and the American Joint Committee on Cancer (AJCC) [11, 12]. These guidelines emphasize the importance of evaluating PNI in CRC specimens, as it provides valuable prognostic information and aids in treatment decision-making.

### Patient selection and matched variables

**Fig. 1:**
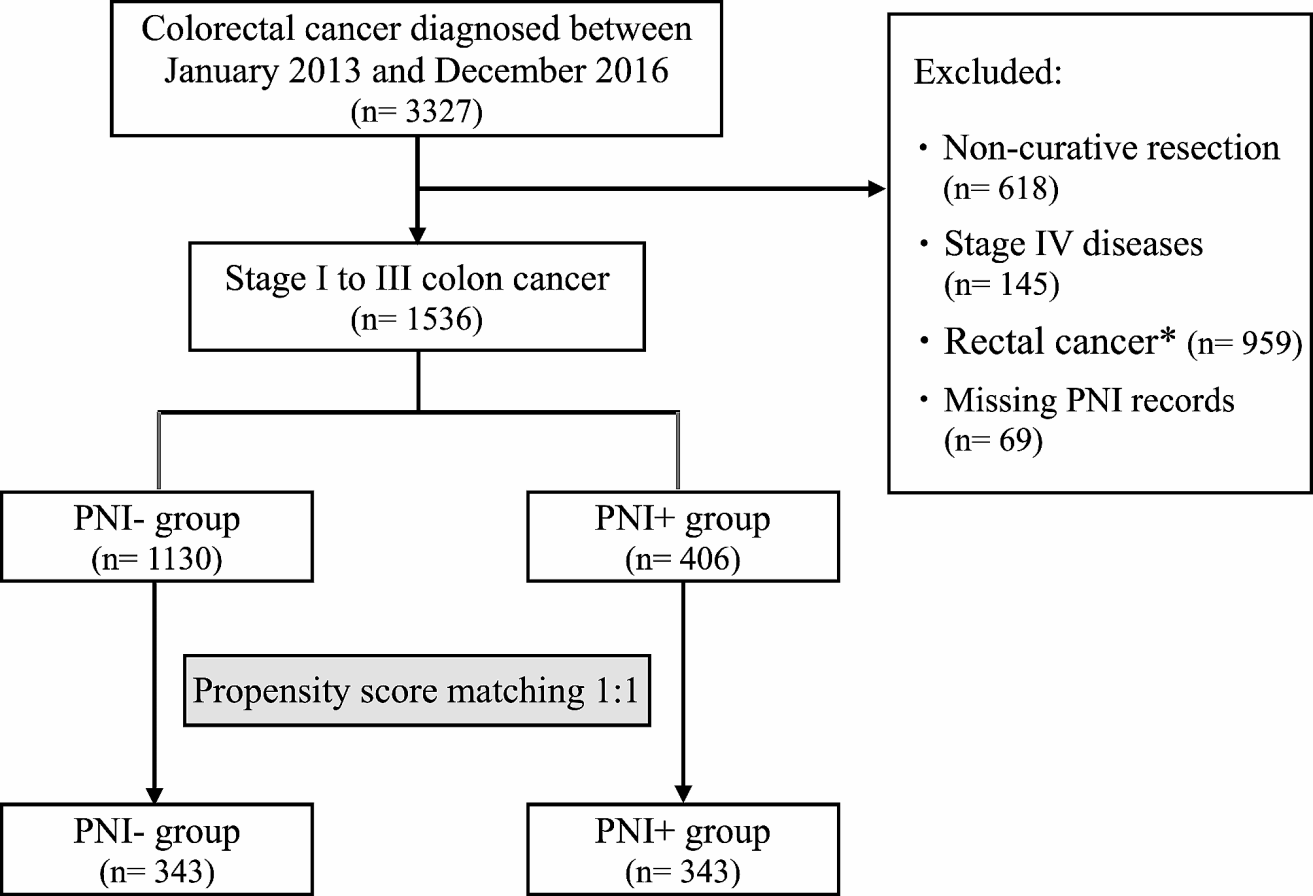
Flowchart of patient selection. *Tumors within 12 cm of the anal verge are classified as rectal cancers. Abbreviations: PNI-, absence of perineural invasion; PNI+, presence of perineural invasion

The present study included 3,327 patients who underwent radical resection for CRC from January 2013 to December 2016. Among these 3,327 patients, we excluded 618 who had undergone noncurative surgery, 145 who had stage IV disease, 959 who were diagnosed with rectal cancer, and 69 for whom PNI data were missing. Consequently, 1,536 patients remained eligible for inclusion in the study, including 1,130 in the PNI-negative (PNI-) group and 406 in the PNI-positive (PNI+) group (Fig. 1).

### Propensity score matching

Propensity score matching (PSM) was implemented in a 1:1 ratio to mitigate selection bias caused by evident disparities in sample sizes and unevenly distributed covariates. This was managed by using a match tolerance of 0.001. The factors included in PSM included age, sex, carcinoembryonic antigen (CEA) level, tumor site, pathological T stage (pT stage), pathological N stage (pN stage), and administration of adjuvant chemotherapy. According to the 1:1 PSM procedure, each group comprised 343 patients (Fig. 1).

### Follow-up and measurement outcomes

Physicians within the same department at this institution adhered to standardized follow-up procedures and an adjuvant treatment protocol. The actual stage of the disease was meticulously evaluated at a weekly multidisciplinary team meeting based on clinical data and pathology reports. Nevertheless, the final determination regarding implementing adjuvant chemotherapy depended on the clinician’s opinion and the patient’s informed decision.

In addition, all patients underwent a comprehensive postoperative follow-up regimen. The program involved regular outpatient appointments, commonly scheduled at intervals of 3 to 6 months, during which patients received physical examinations and CEA tests. The follow-up strategy also included colonoscopies, chest X-rays, and abdominal sonography or abdominal computed tomography scans at varying intervals following surgery. This comprehensive approach ensured thorough monitoring of treatment and disease progression.

The primary study outcomes included cancer recurrence and long-term survival. Long-term results were assessed by examining disease-free survival (DFS) and overall survival (OS). The first instance of cancer recurrence was defined as the date that either local recurrence or distant metastases were confirmed through histological analysis of biopsy specimens, additional surgical interventions, or radiographic studies. DFS was defined as the duration between cancer resection and the date of the first recurrence, mortality, or the end of the last follow-up. OS was defined as the duration between the time of cancer resection and either mortality or the last follow-up date.

### Regimen of adjuvant chemotherapy

During the research period, chemotherapy was initiated for patients between three weeks and two months after surgery. Adjuvant chemotherapy was generally advised for patients who presented with pathological stage II and exhibited high-risk characteristics, including T4 tumor, lymphovascular invasion, clinical obstruction, or stage III. The surgeon or oncologist chooses the chemotherapy regimen based on the patient’s unique situation and preferences. The available treatment choices were either oral tegafur/uracil (UFT) plus leucovorin for six months to a year, capecitabine given over eight cycles, or FOLFOX (fluorouracil, leucovorin, and oxaliplatin) given over twelve cycles.

### Statistical analysis

Statistical analyses were conducted using SPSS Statistics version 22.0 (IBM, Armonk, NY, USA). The χ2 test was used to compare the frequencies and proportions of categorical factors that were used to show clinicopathological characteristics. Student’s t test was used to assess continuous variables, which are reported as the means and standard deviations. We applied 1:1 PSM to address potential selection bias and control for confounding variables and conducted multivariate logistic regression analyses incorporating several covariates. The log-rank test was used to evaluate differences between DFS, OS, and time-to-event probability, which were computed and visualized using the Kaplan‒Meier technique. All statistical tests in this study were two-tailed, and *p* < 0.05 was considered to indicate statistical significance.

## Results

### Patient characteristics before and after propensity score matching

In this cohort study, a total of 1,536 patients were enrolled, with 1,130 exhibiting PNI-negative tumors and 406 (26.4%) manifesting PNI-positive tumors. Subsequently, 686 patients were meticulously selected for a 1:1 PSM protocol, yielding 343 patients in each group (Fig. 1). Table 1 provides a comprehensive overview of the baseline characteristics of these two matched groups. Prior to PSM, patients with PNI + tumors exhibited a notably higher prevalence of preoperative CEA levels exceeding 5 ng/mL (21.0% vs. 34.5%, *p* < 0.001). Furthermore, the administration of adjuvant chemotherapy was more frequent among PNI + patients (39.1% vs. 70.4%, *p* < 0.001). However, no significant differences were discerned in terms of age, sex, or tumor location between these groups. Following PSM, no appreciable differences between the two groups were observed (Table 1).

**Table 1:** Comparison of clinicopathological features in stage I to III colon cancer patients with and without perineural invasion (PNI) before and after propensity score matching.a

Variable	Before matching				After matching		
	PNI – (*N* = 1130)	PNI + (*N* = 406)	*p*-value		PNI – (*N* = 343)	PNI + (*N* = 343)	*p*-value
**Age, ** *n* ** (%)**			0.305				0.592
Age ≤ 65	576 (51.0%)	219 (53.9%)			178 (51.9%)	185 (53.9%)	
Age > 65	554 (49.0%)	187 (46.1%)			165 (48.1%)	158 (46.1%)	
**Sex, ** *n* ** (%)**			0.173				0.760
Female	515 (45.6%)	201 (49.5%)			171 (49.9%)	175 (51.0%)	
Male	615 (54.4%)	205 (50.5%)			172 (50.1%)	168 (49.0%)	
**Preoperative BMI, kg/m** ^**2**^	24.3 (4.0)	23.9 (3.8)	0.121		24.2 (4.1)	23.9 (3.8)	0.333
**Preoperative CEA level, n (%)**			< 0.001				0.505
CEA Level ≤ 5	886 (79.0%)	262 (65.5%)			244 (71.1%)	236 (68.8%)	
CEA Level > 5	235 (21.0%)	138 (34.5%)			99 (28.9%)	107 (31.2%)	
**Tumor location, ** *n* ** (%)**			0.616				0.532
Right-sided	439 (38.8%)	152 (37.4%)			139 (40.5%)	131 (38.2%)	
Left-sided	691 (61.2%)	254 (62.6%)			204 (59.5%)	212 (61.8%)	
**Adjuvant chemotherapy, ** *n* ** (%)**	439 (39.1%)	285 (70.4%)	< 0.001		238 (69.4%)	239 (69.7%)	0.934
**Histologic type, ** *n* ** (%)**			0.029				0.119
Adenocarcinoma	1064 (94.2%)	382 (94.1%)			316 (92.1%)	323 (94.2%)	
Mucinous adenocarcinoma	59 (5.2%)	16 (3.9%)			2 (0.6%)	6 (1.7%)	
Signet ring cell adenocarcinoma	5 (0.4%)	7 (1.7%)			24 (7.0%)	13 (3.8%)	
**Histologic grade, ** *n* ** (%)**			< 0.001				0.596
Well differentiation	151 (13.4%)	14 (3.4%)			14 (4.1%)	12 (3.5%)	
Moderate differentiation	877 (77.6%)	339 (83.5%)			294 (85.7%)	288 (84.0%)	
Poor differentiation	97 (8.6%)	53 (13.1%)			35 (10.2%)	43 (12.5%)	
**pT stage, ** *n* ** (%)**			< 0.001				0.749
pT1	225 (19.9%)	4 (1.0%)			3 (0.9%)	4 (1.2%)	
pT2	173 (15.3%)	20 (4.9%)			14 (4.1%)	18 (5.2%)	
pT3	582 (51.5%)	244 (60.1%)			225 (65.6%)	230 (67.1%)	
pT4	150 (13.3%)	138 (34.0%)			101 (29.4%)	91 (26.5%)	
**N stage, ** *n* ** (%)**			< 0.001				0.349
N0	715 (63.3%)	133 (32.8%)			139 (40.5%)	121 (35.3%)	
N1	276 (24.4%)	156 (38.4%)			128 (37.3%)	136 (39.7%)	
N2	139 (12.3%)	117 (28.8%)			76 (22.2%)	86 (25.1%)	
**Examined lymph node number**			0.044				0.734
< 12	39 (3.5%)	6 (1.5%)			4 (1.2%)	5 (1.5%)	
≥ 12	1090 (96.5%)	399 (98.5%)			339 (98.8%)	337 (98.5%)	

Tumor location, histologic type and histologic grade were selected first record

Right-sided colon: cecum, A-colon, T-colon; Left-sided colon: splenic-flexure, D-colon, S-colon, rectosigmoid

^a^Matching (1:1) was done with propensity score for age, sex, CEA level, tumor location, pT stage, *N* stage, and adjuvant chemotherapy

### Pathological features before and after propensity score matching

Table 1 shows the pathological features of both groups. Notably, there was a significant discrepancy in the pT stage and pN stage (*p* < 0.001) prior to PSM. Moreover, histologic type, histologic grade, and the number of examined lymph nodes exhibited a pronounced association with the presence of PNI. Remarkably, all disparities between the two groups vanished after the implementation of PSM.

### The prognostic value of PNI for oncological outcomes

**Fig. 2:**
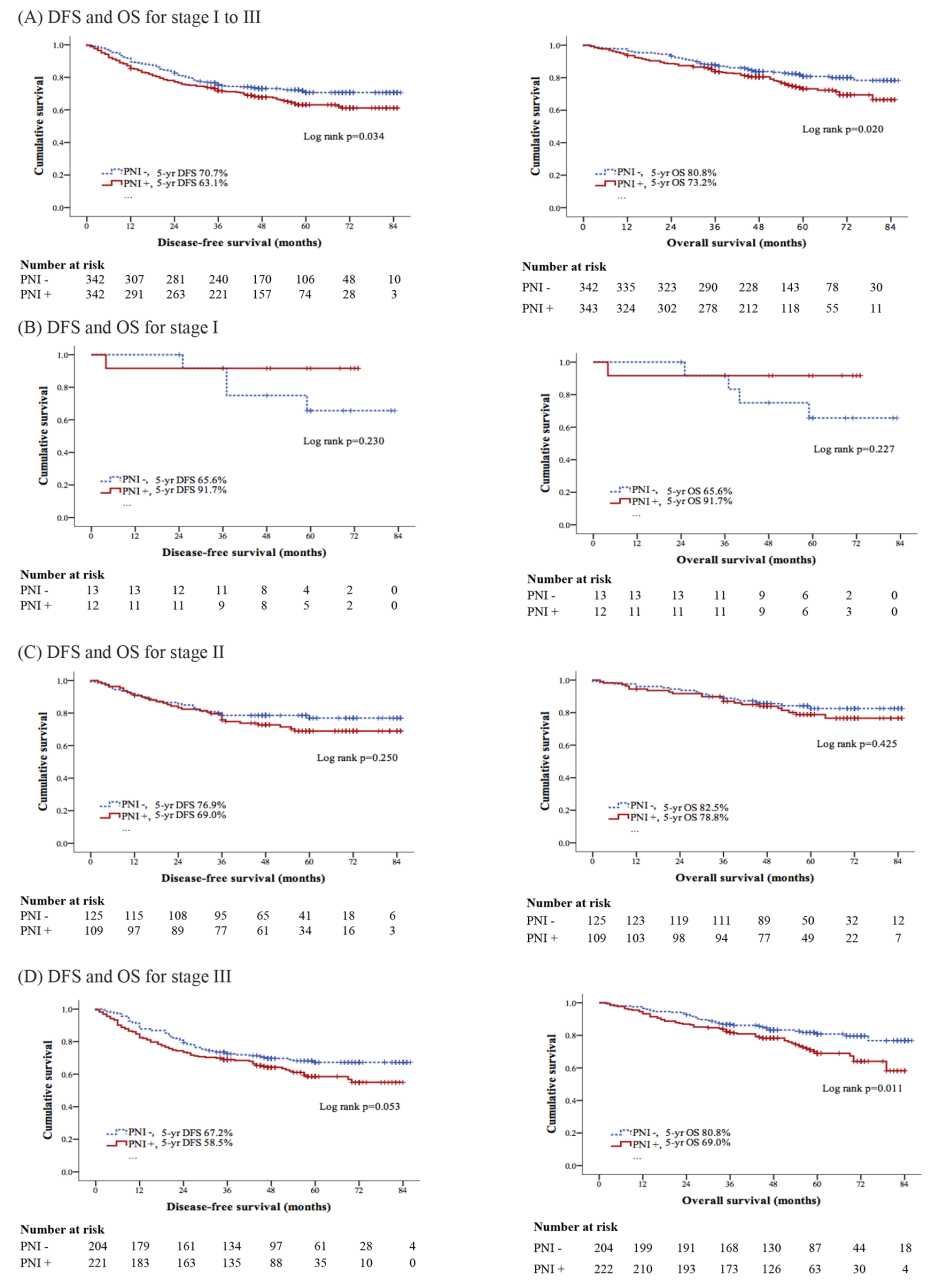
Kaplan-Meier curves of disease-free survival (DFS) and overall survival (OS) with different pTNM stages in colon cancer patients according to the presence of PNI or not. **(A)** DFS and OS for stage I to III (*p* = 0.034 and 0.021 respectively); **(B)** DFS and OS for stage I (*p* = 0.230 and 0.227 respectively); **(C)** DFS and OS for stage II (*p* = 0.250 and 0.470 respectively); **(D)** DFS and OS for stage III (*p* = 0.053 and 0.014 respectively)

Following PSM, the 5-year DFS rate for the PNI- group was 71% versus 63% for the PNI + group in stages I-III. This disparity in the 5-year DFS rate, as well as that in the 5-year OS rate was statistically significant, indicating worse outcomes for colon cancer patients with PNI + tumors than for those with PNI- tumors (*p* = 0.034 and 0.020, respectively) (Fig. 2). Additionally, in stage I, comprising 13 patients without PNI and 12 patients with PNI, neither group exhibited appreciable differences in the DFS rate or OS rate (*p* = 0.230 and *p* = 0.227, respectively). Similarly, in stage II, where the patient count was 126 and 109 for PNI- and PNI + tumors, respectively, neither difference between the two groups was statistically significant (DFS, *p* = 0.250; OS, *p* = 0.425). However, PNI + tumors were associated with significantly lower 5-year DFS rate and 5-year OS rate exclusively in stage III (*p* = 0.053 and *p* = 0.011, respectively), with 204 patients in the PNI- group and 222 in the PNI + group.

### Subgroup analysis for the prognostic value of PNI on oncological outcomes

In an effort to elucidate the association between PNI and significant covariates impacting disease recurrence, we conducted a Cox regression model analysis employing the propensity score-matched data. Table 2 shows that PNI exhibited no significant association with age, sex, preoperative CEA levels, tumor location, histologic type, pT stage, pN stage, lymphovascular invasion, number of examined lymph nodes, or adjuvant chemotherapy in altering the odds of recurrence. Conversely, in poorly differentiated colon cancers, there was a fourfold greater odds of recurrence in the PNI + group than in the PNI- group (OR, 4.15; *p* = 0.016).

**Table 2:** Subgroup analysis for disease recurrence

Variable	PNI – (*N* = 343)	PNI + (*N* = 343)	Odds ratio (95% CI)	*p*-value
**Age**				
Age ≤ 65	37/178 (20.8%)	41/185 (22.2%)	1.09 (0.66–1.79)	0.750
Age > 65	34/165 (20.6%)	42/158 (26.6%)	1.40 (0.83–2.34)	0.206
**Sex**				
Female	39/171 (22.8%)	40/175 (22.9%)	1.00 (0.61–1.66)	0.991
Male	32/172 (18.6%)	43/168 (25.6%)	1.51 (0.90–2.53)	0.120
**Preoperative CEA level, ** *n* ** (%)**				
CEA Level ≤ 5	38/244 (15.6%)	45/236 (19.1%)	1.28 (0.80–2.05)	0.312
CEA Level > 5	33/99 (33.3%)	38/107 (35.5%)	1.10 (0.62–1.96)	0.742
**Tumor location, ** *n* ** (%)**				
Right-sided	24/139 (17.3%)	30/131 (22.9%)	1.42 (0.78–2.59)	0.247
Left-sided	47/204 (23.0%)	53/212 (25.0%)	1.11 (0.71–1.75)	0.640
**Histologic type, ** *n* ** (%)**				
Adenocarcinoma	66/316 (20.9%)	77/323 (23.8%)	1.19 (0.82–1.72)	0.371
Mucinous adenocarcinoma	5/24 (20.8%)	5/13 (38.5%)	2.38 (0.54–10.5)	0.249
**Histologic grade, ** *n* ** (%)**				
Well to moderate differentiation	67/308 (21.8%)	68/300 (22.7%)	1.05 (0.72–1.55)	0.786
Poor differentiation	4/35 (11.4%)	15/43 (34.9%)	4.15 (1.23-14.0)	0.016
**pT stage, ** *n* ** (%)**				
pT1-2	1/17 (5.9%)	0/22 (0%)	NA	0.249
pT3	36/225 (16.0%)	49/230 (21.3%)	1.42 (0.88–2.29)	0.147
pT4	34/101 (33.7%)	34/91 (37.4%)	1.18 (0.65–2.13)	0.593
**N stage, ** *n* ** (%)**				
N0	20/139 (14.4%)	18/121 (14.9%)	1.04 (0.52–2.07)	0.912
N1	25/128 (19.5%)	31/136 (22.8%)	1.22 (0.67–2.20)	0.517
N2	26/76 (34.2%)	34/86 (39.5%)	1.26 (0.66–2.39)	0.484
**Lymphovascular invasion, ** *n* ** (%)**				
no	40/246 (16.3%)	30/180 (16.7%)	1.03 (0.61–1.73)	0.911
yes	31/97 (32.0%)	53/163 (32.5%)	1.03 (0.60–1.76)	0.926
**Examined lymph node number**				
< 12	0/4 (0%)	3/5 (60.0%)	0.33 (0.11–1.03)	0.058
≥ 12	71/339 (20.9%)	80/337 (23.7%)	1.18 (0.82–1.69)	0.383
**Adjuvant chemotherapy, ** *n* ** (%)**				
no	16/105 (15.2%)	19/104 (18.3%)	1.24 (0.60–2.58)	0.557
yes	55/238 (23.1%)	64/239 (26.8%)	1.22 (0.80–1.84)	0.354

Abbreviations: CI, confidence interval

### The predictive value of PNI for oncological outcomes

**Fig. 3:**
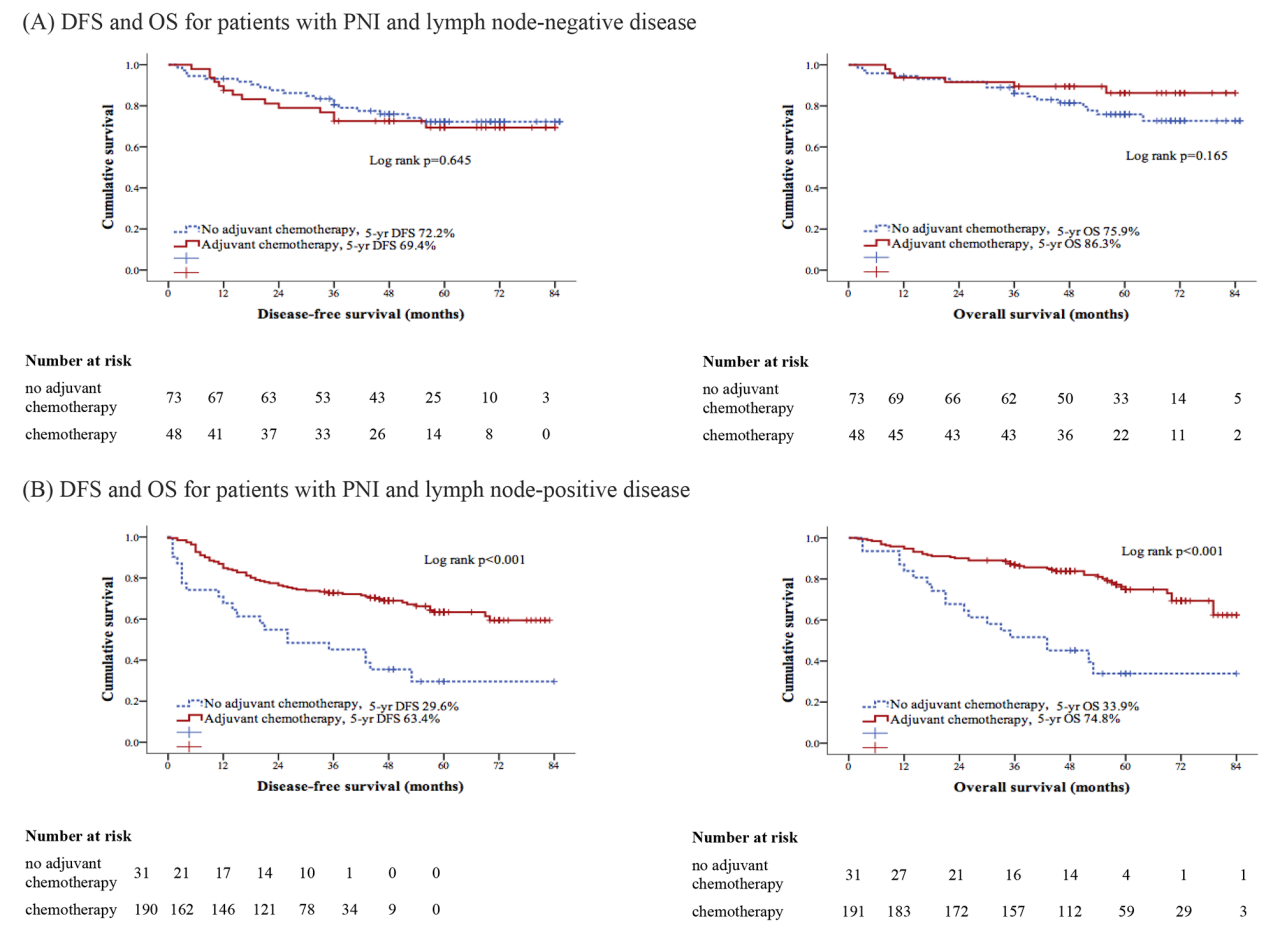
Kaplan-Meier curves for disease-free survival (DFS) and overall survival (OS) in patients with PNI and complete tumor resection. According to adjuvant chemotherapy status. **(A)** DFS and OS for patients with PNI and lymph node-negative disease (*p* = 0.645 and 0.165 respectively); **(B)** DFS and OS for patients with PNI and lymph node-positive disease (both *p* < 0.001)

To examine the predictive value of PNI for the response to adjuvant chemotherapy, a subsequent Cox regression model analysis was conducted. Figure 3 illustrates that adjuvant chemotherapy did not exert a significant impact on DFS or OS among patients with PNI + tumors and node-negative disease (*p* = 0.645 and *p* = 0.165, respectively), with 48 patients in the adjuvant chemotherapy group and 73 in the nonadjuvant chemotherapy group. In contrast, adjuvant chemotherapy significantly enhanced the DFS and OS among patients with PNI + tumors and node-positive disease (both *p* < 0.001), with 191 patients in the adjuvant chemotherapy group and 31 in the nonadjuvant chemotherapy group.

We conducted a Cox proportional hazards regression analysis for DFS and OS among stage I to III colon cancer patients utilizing data collected prior to PSM. This analytical approach allows for the adjustment of potential confounders and facilitates a more comprehensive understanding of the relationship between adjuvant chemotherapy, PNI, N status, and long-term survival outcomes. The outcomes of the Cox proportional hazards regression analysis are delineated in Supplementary Table 1, which is accessible in the Supplementary Material section.

In addition to the main analyses, we also performed Kaplan-Meier curves to assess DFS and OS in a subgroup of patients with PNI-negative tumors who underwent complete tumor resection, stratified by adjuvant chemotherapy status. The results of these analyses are presented in Figure S1, which can be found in the Supplementary Material section.

## Discussion

Propensity score matching was utilized in this extensive cohort study, comprising 1,536 patients diagnosed with colon cancer, to assess the influence of PNI on a number of patient characteristics, pathological features, and oncological results. PNI + tumors were correlated with elevated preoperative CEA levels and increased adjuvant chemotherapy administration before matching. After matching, however, these disparities were mitigated. Pathological characteristics that were previously different between groups, such as pT stage, pN stage, histologic type, and grade, were balanced after matching. According to the study, PNI + tumors were associated with lower 5-year DFS and OS rates in stages I–III, which was especially significant for poorly differentiated colon cancers. PNI exhibited no significant association with covariates that influence disease recurrence, except for poorly differentiated malignancies, where the odds increased by a factor of four. Furthermore, depending on the lymph node status, the predictive value of PNI for the response to adjuvant chemotherapy, highlighting the complexity of the influence of this factor on treatment outcomes. This study elucidates the complex relationship between PNI and the prognosis of colon cancer, making it a valuable resource for comprehending its clinical consequences and developing customized treatment plans.

Several initial variables were incorporated into our study: age, sex, preoperative body mass index (BMI), CEA level, tumor site, histologic type or grade, pT stage, pN stage, and adjuvant chemotherapy administration. Significant correlations were found between PNI and age, sex, CEA level, tumor site, pT stage, pN stage, and adjuvant chemotherapy treatment. However, the PSM procedure ultimately disregarded the association of these factors with PNI. Additionally, our results showed that PNI has a negative effect on both the 5-year DFS and OS rates in colon cancer patients. After the patients were grouped into stages I, II, and III disease, the presence of PNI was found to be significantly associated with a lower 5-year OS rate (*p* = 0.011) exclusively among stage III patients. Although the 5-year DFS rate (*p* = 0.053) did not attain statistical significance, it is noteworthy that p-values falling between 0.05 and 0.10 are classified as “marginally significant” [13]. Hence, the observed decrease in the 5-year DFS rate (*p* = 0.053) among stage III patients with PNI retains some relevance and should not be entirely disregarded. And no significant correlations were found in stage I or II. These findings are consistent with several retrospective review studies that found no discernible changes in OS rates between stage II colorectal cancer patients with and without PNI positivity [2, 7]. On the other hand, some studies have highlighted the importance of PNI in terms of its predictive significance [3, 4, 6, 8, 14–17]. In 13,528 stage II colon cancer patients, for instance, Mirkin et al. found that PNI was an independent detrimental prognostic factor for OS [7]. Similarly, Kang et al. proposed that the combination of T4 tumor, lymphovascular invasion, and PNI predicted worse recurrence-free survival in stage II-III colon cancer patients [16]. These complicated results highlight the significance of PNI in determining the prognosis of colon cancer and the need to consider disease stage and other factors when choosing treatment.

Existing data previously identified various prognostic variables influencing disease recurrence in colorectal cancer patients undergoing curative resection [18–20]. PNI has been linked to higher recurrence rates in stage I–III colorectal cancer patients treated with curative intent, according to a study by Holt et al. [18]. Nevertheless, there is an apparent lack of information in the literature regarding the exact association between PNI and other significant variables influencing the recurrence of the disease. We conducted a subgroup analysis to address this knowledge gap and identified a significantly greater likelihood of recurrence in poorly differentiated colon cancers among the PNI + group than among the PNI- group. The observed association highlights the complex interaction that poorly differentiated tumors and PNI have with disease recurrence. More research is needed to clarify the mechanisms and biological factors contributing to this connection, which will help us better grasp the prognostic significance of PNI in colorectal cancer.

Patients with stage III CRC have been shown to benefit from postoperative adjuvant chemotherapy. Nevertheless, its applicability to stage II CRC patients who have undergone resection remains a subject of ongoing controversy [3]. The direct effect of PNI on CRC patient outcomes is still unclear in the scientific literature. Clarifying the role of PNI in this situation is essential for informing clinical decisions and optimizing treatment strategies for CRC patients. Our study did not confirm the predictive character of PNI in node-negative colon cancer. That is, the results of the present study may support the argument that patients with PNI + tumors and node-negative disease are not candidates for postoperative chemotherapy. Similar to our results, Leijssen, L. G. J., et al. and Peng, Sze-Lin et al. demonstrated that patients with node-negative disease did not significantly benefit from adjuvant chemotherapy when PNI was detected [3, 10, 21]. However, our findings are in contrast with some other reports. In a subgroup analysis conducted by Kang, J. H., et al., adjuvant chemotherapy improved recurrence-free survival in stage II colon cancer with PNI [13]. Another national cancer database study conducted by Alotaibi, A. M., et al. reported that adjuvant chemotherapy was associated with overall survival benefits in stage II colon cancer with high risk factors, including PNI [17]. In addition, two meta-analyses showed that administration of chemotherapy improved DFS in stage II colorectal patients [4, 9].

The inclusion of rectal cancer, however, may introduce additional confounding factors. Neoadjuvant therapy has become a commonly employed treatment approach for individuals with rectal cancer. According to a meta-analysis, patients with rectal cancer who did not receive neoadjuvant therapy were more likely to be PNI+ [4]. Moreover, the literature emphasizes that rectal cancer exhibits a greater propensity for disease recurrence than colon cancer [22]. Kim et al. suggested that PNI is a valuable indicator of recurrence in rectal cancer patients receiving neoadjuvant chemotherapy. This highlights the need for more research to understand how PNI works and its full effects during neoadjuvant chemotherapy [23]. Our study was limited to colon cancer to reduce any biases that may have been caused by neoadjuvant therapy.

Adjuvant chemotherapy is the recommended treatment for stage III colon cancer patients after curative resection [24]. Consistent with earlier research [7, 15], our study results showed that PNI is most helpful in predicting survival in stage III cancer. Patients with PNI + malignancies who did not undergo adjuvant chemotherapy exhibited significantly inferior DFS and OS. We also found that adjuvant treatment significantly affected PNI + tumors and node-positive disease, substantially lowering the risk of death. This highlights the critical role adjuvant chemotherapy plays in reducing the unfavorable prognostic consequences linked to PNI in a specific subset of patients with colon cancer.

In our study, the results suggest that patients with PNI + tumors and node-negative disease may not benefit from adjuvant chemotherapy. However, we acknowledge that several previous studies have reported an association between adjuvant chemotherapy and improved prognosis in stage II colon cancer patients with high-risk factors, including PNI. To address this discrepancy, we propose several potential explanations for the observed differences in results.

Firstly, tumor biology, including molecular and genetic characteristics, may play a significant role in determining the response to adjuvant chemotherapy in stage II colon cancer patients with PNI. For example, microsatellite instability (MSI) status and other molecular markers have been shown to influence prognosis and treatment response in CRC [25, 26]. Differences in the prevalence of these molecular features among study populations could contribute to varying conclusions regarding the benefit of adjuvant chemotherapy in patients with PNI.

Secondly, patient comorbidities and overall health status may also impact the effectiveness of adjuvant chemotherapy in stage II colon cancer patients with high-risk factors. Patients with significant comorbidities or poor performance status may be less likely to tolerate or benefit from adjuvant chemotherapy, which could influence the observed outcomes in different studies [27, 28]. Variations in the inclusion criteria and baseline characteristics of study populations could therefore contribute to differing conclusions regarding the role of adjuvant chemotherapy in this patient subgroup.

The study had several limitations that warrant consideration in interpreting the findings. First, its retrospective design, confined to a single-center setting, raises concerns regarding the generalizability of the results to broader populations. Variability in patient demographics, treatment modalities, and institutional practices introduces potential limitations to the external validity of our conclusions. Despite applying PSM to address baseline imbalances, it is crucial to acknowledge the possibility of residual confounding factors persisting in our analysis. Moreover, the lack of essential genetic features in our dataset, including KRAS and BRAF mutation status and MSI status, represents a notable limitation. These genetic factors hold established prognostic significance in colon cancer [29–31], and their exclusion hinders a comprehensive understanding of the molecular landscape and its direct implications for patient outcomes. Our study predominantly focused on clinical and pathological factors, lacking detailed information on patient comorbidities, overall health status, lifestyle factors, and socioeconomic status. This omission limits the comprehensive exploration of the multifaceted influences on outcomes, potentially overlooking crucial determinants of patient prognosis. Furthermore, one of the limitations of our study is the relatively small sample size, particularly for stage I tumors, as depicted in Fig. 2. Following PSM, there were only 13 patients with stage I tumors in the PNI- group and 12 in the PNI + group. This restricted sample size may impact the statistical power and our ability to draw definitive conclusions regarding the prognostic significance of PNI in stage I colon cancer. Consequently, the sample size of the present study may have also constrained its statistical power to discern a significant difference. Hence, it is imperative to interpret this marginally significant result with caution and regard it as a potential indication of a clinically significant difference warranting further investigation.

In light of these limitations, a cautious interpretation of our findings is warranted. Future research endeavors should address these intricacies through prospective, multicenter studies incorporating a broader array of variables to enhance the robustness and applicability of our conclusions.

## Conclusion

In conclusion, our propensity score-matched cohort study of 1,536 colon cancer patients shows that PNI + tumors are significantly linked to adverse outcomes, as demonstrated by marginally significant lower 5-year DFS and significant lower OS rates in stages III. Specifically, poorly differentiated tumors show a more vital association between PNI and disease progression. Furthermore, the study reveals exciting complexities in the predictive value of PNI for adjuvant chemotherapy response, demonstrating variations according to nodal status. These findings highlight the complicated influence of PNI on colon cancer prognosis and underline the necessity of tailored treatment strategies that consider PNI status. Future research should focus on the underlying molecular mechanisms underlying the correlation between aggressive tumor behavior and PNI, especially in poorly differentiated malignancies. Investigating the molecular landscape, including genetic characteristics, may yield important information on the mechanisms behind the effects of PNI.

### Electronic supplementary material

Below is the link to the electronic supplementary material.

Supplementary Material 1

Supplementary Material 2

## Data Availability

No datasets were generated or analysed during the current study.

## Abbreviations

CRC: Colorectal cancer
PNI: Perineural invasion
PSM: Propensity score matching
PNI+: PNI-positive
PNI-: PNI-negative
DFS: Disease-free survival
OS: Overall survival
CEA: Carcinoembryonic antigen
pT stage: Pathological T stage
pN stage: Pathological N stage
BMI: Body mass index
MSI: Microsatellite instability
