# Supplementary material for: The prognostic and predictive significance of perineural invasion in stage I to III colon cancer: a propensity score matching-based analysis
Source: World J Surg Oncol. 2024 May 11;22:129. doi: 10.1186/s12957-024-03405-6 (PMC11088143; doi:10.1186/s12957-024-03405-6)
Supplement: Supplementary file 1 — Supplementary Material 1 [file 12957_2024_3405_MOESM1_ESM.docx]

**Supplementary Table 1.** Cox proportional hazards regression analysis for disease-free survival (DFS) and overall survival (OS) in stage I to III colon cancer patients.

| **Demographics and clinical features** | **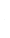Disease-free survival** | | |  | **Overall survival** | | |
| --- | --- | --- | --- | --- | --- | --- | --- |
|  | **Univariate p value** | **Multivariate HR (95% CI)** | **P value** |  | **Univariate p value** | **Multivariate HR (95% CI)** | **P value** |
| **Age** | <0.001 |  | <0.001 |  | <0.001 |  | <0.001 |
| ≤65 |  | 1.00 |  |  |  | 1.00 |  |
| >65 |  | 1.60 (1.29-1.98) |  |  |  | 2.26 (1.73-2.96) |  |
| **Sex** | 0.215 |  |  |  | 0.326 |  |  |
| Female |  |  |  |  |  |  |  |
| Male |  |  |  |  |  |  |  |
| **Preoperative BMI** | 0.055 |  | 0.317 |  | 0.001 |  | 0.017 |
| <25 |  | 1.00 |  |  |  | 1.00 |  |
| ≥25 |  | 0.90 (0.73-1.11) |  |  |  | 0.73 (0.57-0.95) |  |
| **Preoperative CEA level** | <0.001 |  | <0.001 |  | <0.001 |  | <0.001 |
| ≤5 |  | 1.00 |  |  |  | 1.00 |  |
| >5 |  | 1.76 (1.43-2.18) |  |  |  | 1.80 (1.40-2.31) |  |
| **Tumor location** | 0.962 |  |  |  | 0.074 |  |  |
| Right-sided |  |  |  |  |  |  |  |
| Left-sided |  |  |  |  |  |  |  |
| **Adjuvant chemotherapy** | 0.005 | 0.66 (0.52-0.85) | 0.001 |  | 0.063 | 0.42 (0.32-0.57) | <0.001 |
| **Histologic type** | <0.001 |  | 0.001 |  | <0.001 |  | <0.001 |
| Adenocarcinoma |  | 1.00 |  |  |  | 1.00 |  |
| Mucinous adenocarcinoma |  | 0.84 (0.27-2.62) |  |  |  | 1.40 (0.45-4.41) |  |
| Signet ring cell adenocarcinoma |  | 0.99 (0.65-1.51) |  |  |  | 1.18 (0.72-1.92) |  |
| **Histologic grade** | 0.595 |  |  |  | 0.122 |  |  |
| Well to moderate differentiation |  |  |  |  |  |  |  |
| Poor differentiation |  |  |  |  |  |  |  |
| **Perineural invasion** | <0.001 | 1.26 (1.02-1.57) | 0.036 |  | <0.001 | 1.40 (1.08-1.81) | 0.011 |
| **pT stage** | <0.001 |  | <0.001 |  | <0.001 |  | 0.005 |
| pT1 |  | 1.00 |  |  |  | 1.00 |  |
| pT2 |  | 1.60 (0.92-2.77) |  |  |  | 1.26 (0.70-2.30) |  |
| pT3 |  | 2.23 (1.39-3.57) |  |  |  | 1.66 (1.01-2.73) |  |
| pT4 |  | 3.44 (2.07-5.73) |  |  |  | 2.41 (1.38-4.19) |  |
| **N stage** | <0.001 |  | <0.001 |  | <0.001 |  | <0.001 |
| N0 |  | 1.00 |  |  |  | 1.00 |  |
| N1 |  | 1.65 (1.26-2.15) |  |  |  | 1.71 (1.24-2.34) |  |
| N2 |  | 2.64 (1.98-3.53) |  |  |  | 2.82 (2.00-3.99) |  |
| **Examined lymph node number** | 0.682 |  |  |  | 0.751 |  |  |
| <12 |  |  |  |  |  |  |  |
| ≥12 |  |  |  |  |  |  |  |

Tumor location, histologic type and histologic grade were selected first record.

Right-sided colon: cecum, A-colon, T-colon; Left-sided colon: splenic-flexure, D-colon, S-colon, rectosigmoid

*CI* confidence interval; *HR* hazard ratio
