# Supplementary material for: The prognostic and predictive significance of perineural invasion in stage I to III colon cancer: a propensity score matching-based analysis
Source: World J Surg Oncol. 2024 May 11;22:129. doi: 10.1186/s12957-024-03405-6 (PMC11088143; doi:10.1186/s12957-024-03405-6)
Supplement: Supplementary file 2 — Supplementary Material 2 [file 12957_2024_3405_MOESM2_ESM.docx]

(A) DFS and OS for patients with PNI-negative and lymph node-negative disease


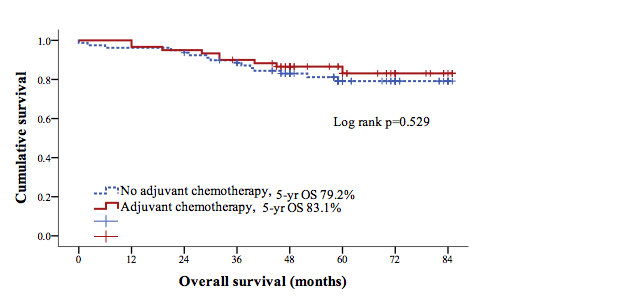

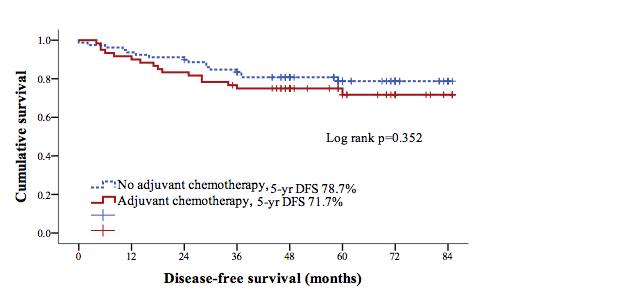


| **Number at risk** | |  |  |  |  |  |  |  |
| --- | --- | --- | --- | --- | --- | --- | --- | --- |
| No adjuvant chemotherapy | 79 | 74 | 70 | 62 | 45 | 25 | 11 | 1 |
| Chemotherapy | 60 | 54 | 50 | 44 | 28 | 20 | 9 | 5 |

| **Number at risk** | |  |  |  |  |  |  |  |
| --- | --- | --- | --- | --- | --- | --- | --- | --- |
| No adjuvant chemotherapy | 79 | 76 | 73 | 65 | 46 | 25 | 11 | 1 |
| Chemotherapy | 60 | 58 | 57 | 53 | 31 | 21 | 10 | 5 |

(B) DFS and OS for patients with PNI-negative and lymph node-positive disease


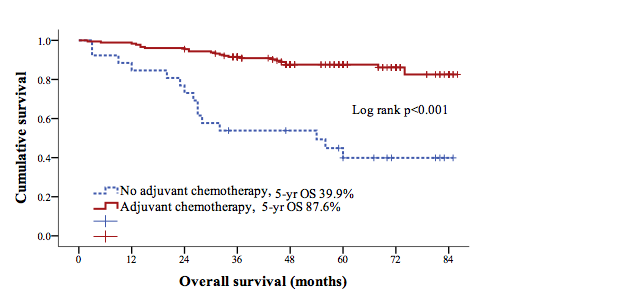

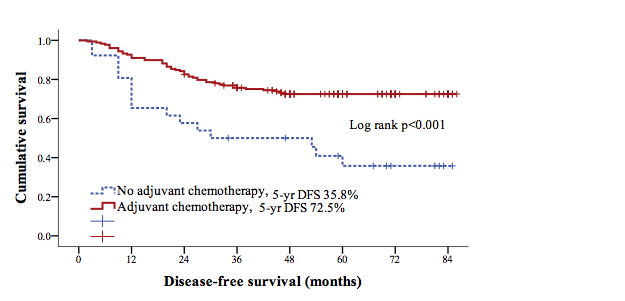


| **Number at risk** | |  |  |  |  |  |  |  |
| --- | --- | --- | --- | --- | --- | --- | --- | --- |
| No adjuvant chemotherapy | 26 | 22 | 19 | 13 | 12 | 7 | 4 | 1 |
| Chemotherapy | 178 | 175 | 169 | 145 | 101 | 65 | 28 | 3 |

| **Number at risk** | |  |  |  |  |  |  |  |
| --- | --- | --- | --- | --- | --- | --- | --- | --- |
| No adjuvant chemotherapy | 26 | 17 | 15 | 12 | 11 | 7 | 4 | 1 |
| Chemotherapy | 178 | 162 | 146 | 122 | 86 | 54 | 24 | 3 |

**Supplementary Figure 1.** Kaplan-Meier curves for disease-free survival (DFS) and overall survival (OS) in patients with PNI-negative and complete tumor resection. According to adjuvant chemotherapy status. (A) DFS and OS for patients with PNI-negative and lymph node-negative disease (p=0.352 and 0.529 respectively); (B) DFS and OS for patients with PNI-negative and lymph node-positive disease (both p<0.001).
